# The Diseases of Sedentary and Advanced Life

**Published:** 1886-05

**Authors:** 


**The Diseases of Sedentary and Advanced Life.** A work for medical and lay readers. By I. MILNER FOTHERGILL, M. D., Physician to the London Hospital, etc., etc. New York: D. Appleton & Co.

If Fothergill's writings are not always profound, they are at least always interesting, and, under a sparkling style, he often imparts more knowledge and brings to bear more practical common sense in his teaching than others. The present work is really a valuable one. It covers a ground which is usually but lightly touched upon by the ordinary text-book. It is full of valuable suggestions, and either the old or young physician who may take it up will read it through with interest and profit.

The Appletons' have presented it in very handsome dress. The book deserves, and we think will have, a large sale.

